# Revealing the inner workings of organoids

**DOI:** 10.15252/embj.201796860

**Published:** 2017-04-24

**Authors:** Cristina Dias, François Guillemot

**Affiliations:** ^1^ The Francis Crick InstituteLondonUK

**Keywords:** Development & Differentiation

## Abstract

The mechanisms by which human stem cells self‐organise into brain‐like tissues in 3D organoid culture are poorly understood. In this issue of *The EMBO Journal*, Renner and Lancaster *et al* demonstrate that in the absence of external stimuli, human cerebral organoids develop large forebrain structures that display specific landmarks of spatial and temporal patterning, including signalling centres producing known morphogens. The generation of cerebral organoids is therefore likely to reflect normal brain development.

Characterisation of the intricate processes underlying human brain development is pivotal for our understanding of the unique properties of this organ and elucidating developmental and degenerative disease processes. The brain of humans is extraordinarily more complex than that of commonly used mammalian models in size, shape, neuronal density, diversity of cell types and gyration. Because of this divergence, pathologies are frequently not recapitulated in model organisms. Studies of human foetal brain have been valuable in defining human‐specific temporal and spatial cortical developmental programmes (Pollen *et al*, 2015). However, these approaches are limited by the need to access foetal tissue samples, impracticality of genetic manipulation and long‐term culture. Hence, cell culture models of human brain development are needed to address developmental questions and modelling of disease.

Initial efforts to develop *in vitro* models of human brain development focused on the intrinsic capacity of human pluripotent cells to differentiate into neurons of the cerebral cortex when cultured in the absence of added morphogens. Such cultures successfully produce neurons of different cortical layer identities (e.g. Gaspard *et al*, 2008) but because of their two‐dimensional organisation, they do not recapitulate the complex cytoarchitecture of the developing human brain. The work from Yoshiki Sasai's group thus represented a breakthrough: they showed that when cortical tissue generated from pluripotent stem cells by modulating specific signalling pathways was grown in suspension, it self‐organised into three‐dimensional (3D) structures comprising distinct progenitor and neuronal layers and recapitulating early stages of cortical development (Eiraku *et al*, 2008). Subsequent studies showed that such 3D structures contain outer radial glial cells, a prominent progenitor population that appears relatively late in human cortical development (Kadoshima *et al*, 2013), as well as astrocytes and mature neurons that establish functional synapses (Paşca *et al*, 2015). These organoids can thus be used to model pathologies, such as autism spectrum disorder, and study disease mechanisms previously elusive in the mouse (Mariani *et al*, 2015; Bershteyn *et al*, 2017).

However, such 3D cortical structures present certain limitations that restrict their use to model human brain development, including small sizes and paucity of neuronal types other than cortical excitatory neurons. The Knoblich group provided an important advance to the technology by introducing a spinning bioreactor to facilitate oxygen and nutrient absorption, and removing exogenous patterning factors from the medium, leading to the generation of larger and more complex structures (Lancaster *et al*, 2013). Interestingly, these cerebral organoids (COs) contain a broader range of neuronal types than 3D structures generated with previous protocols, with neurons grouped in spatially segregated territories resembling different embryonic brain regions, including cerebral cortex, ventral forebrain, hindbrain and retina. Moreover, ventral forebrain territories produce cortical interneurons that appear to migrate into the cortical regions, as they normally do during forebrain development.

An extraordinary feature of COs is their broad capacity to self‐organise, resulting in the generation of complex structures encompassing multiple brain regions. However, organoids have variable shapes very unlike that of a brain, and the diverse brain tissues they contain appear randomly distributed. It was therefore unclear whether the subdivision of COs into regions of different identities results from patterning events that bear a resemblance to normal developmental mechanisms or represents instead uncoordinated events irrelevant to brain development. To elucidate the mechanisms driving this self‐organisation, Renner and Lancaster *et al* in this issue of *The EMBO Journal* have further characterised COs, focusing on forebrain regions (Renner *et al*, 2017). Because COs lack body axes and well‐defined morphologies that help to recognise the major subdivisions of the embryonic brain, they used a large battery of markers of brain regional identities. They showed the presence of large continuous tissues containing well‐defined neuronal domains and progenitor domains with adjacent ventricular and subventricular zones, which they recognised as cerebral cortex (Fig 1C). They also frequently identified choroid plexus and lateral ganglionic eminence tissue, while hippocampus and medial ganglionic eminence tissue were observed less frequently.

**Figure 1:**
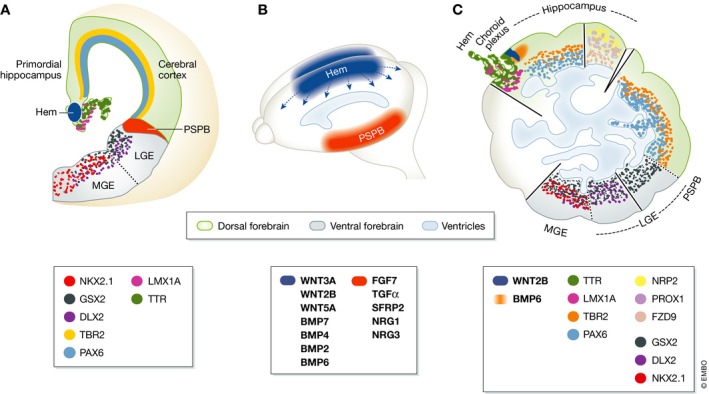
Regional patterning of developing brain is recapitulated in organoids (A) Coronal section of the developing mouse telencephalon (reviewed in Martynoga *et al*, 2012) showing gene expression patterns for markers used by Renner and Lancaster *et al* to define regional identities in human cerebral organoids (Renner *et al*, 2017). PSPB, pallial‐subpallial boundary; LGE, lateral ganglionic eminence; MGE, medial ganglionic eminence. (B) Signalling centres in the mouse telencephalon. Wnt and bone morphogenetic proteins are secreted by a dorso‐medial organising centre, the cortical hem (blue). The PSPB (orange) is a candidate signalling centre expressing several soluble factors, including a WNT inhibitor and FGF, EGF and NRG family ligands. Signals emerging from other forebrain signalling centres are not represented. (C) Diagram of different regions identified by Renner and Lancaster *et al* in concurring and/or different cerebral organoids and recapitulating forebrain patterning. PAX6 and TBR2 identify the ventricular and subventricular zones of the cerebral cortex. The choroid plexus (CP) is defined by its characteristic morphology and expression of TTR and LMX1A. A cortical hem‐like structure, identified by its location between cortical and CP tissue and expression of LMX1A, displays a narrow stripe of WNT2B expression that overlaps with a more diffuse domain of BMP6 expression. Hippocampus is recognised by co‐expression of PROX1, FZD9 and NRP2 and cortical markers PAX6 and TBR2. A sharp interface between TBR2‐expressing cortex and GSX2‐expressing ganglionic eminence marks the presence of a PSPB‐like region. Co‐expression of GSX2 and DLX2 marks the LGE and NKX2.1 expression marks the MGE.

The cortical hem, which is wedged between the choroid plexus and the cerebral cortex in the embryonic brain and produces multiple signalling molecules including WNTs and BMPs, acts as an important signalling centre in patterning of the adjacent forebrain tissue (Caronia‐Brown *et al*, 2014; Fig 1A and B). Several organoids contained tissue sandwiched between choroid plexus and cerebral cortex, expressing the cortical hem marker LMX1A. Remarkably, this tissue also expressed signalling molecules, including WNT2B in a narrow strip and BMP6 in a broader territory, which is also seen in the cortical hem of mouse embryos (Fig 1A). This finding raises the exciting possibility that hem‐like signalling centres participate in the self‐organisation of COs by patterning adjacent tissues. The authors also suggest that signalling molecules produced by these centres might contribute to CO expansion, as reported for the hem *in vivo* (Caronia‐Brown *et al*, 2014). The pallial–subpallial boundary (PSPB) also produces signalling molecules in the embryonic forebrain (Fig 1A and B) and is proposed to act as a signalling centre during forebrain development. The finding that COs contain TBR2^+^ cortical tissue abutting GSX2^+^ ganglionic eminence tissue therefore suggests that signalling centres other than the hem might be generated in COs and contribute to their extensive tissue diversity.

To further investigate the relationships between different brain regions, Renner and Lancaster *et al* made COs transparent and performed 3D image reconstitution. This revealed that COs contain ventricular networks that are lined by a continuous neuroepithelium and connect different brain regions. Thus, molecules produced by signalling centres could act not only locally but also over long distances by travelling across the COs via the ventricular network.

Focusing on cortical regions, the authors asked whether these territories in COs produce the same range of cell types and in the same temporal order as in the cerebral cortex *in vivo*. They found that Cajal‐Retzius cells, deep layer neurons and superficial layer neurons are indeed present and produced sequentially, similarly to the cortical development *in vivo*. Moreover, organoids produce both astrocytes and oligodendrocytes, and both neurons and glial cells acquire morphologies characteristic of mature cells.

Variability between experiments and between organoids in the same experiment is a concern that is often voiced but has not been rigorously documented. Renner and Lancaster *et al* have now addressed this issue by systematically analysing 104 organoids generated in 22 separate experiments. Four of the experiments generated large dorsal forebrain regions in all organoids, 12 had large forebrain regions in some organoids but not in others, and six experiments did not produce any organoid with large forebrain regions. The large degree of self‐organisation taking place during CO development may contribute to this variability. The absence of exogenous cues during CO development leads to the emergence of territories of distinct identities. When these regions happen to be contiguous, the formation of an interface may favour the generation of signalling centres and result in further patterning of the CO. Adding patterning factors at an early step in the protocol has been shown to result in organoids with more uniform size, shape and regional identity, but at the cost of a loss of the regional diversity and extensive self‐organisation seen in COs (Qian *et al*, 2016). A way forward might be to use emerging microfluidic techniques to direct spatial patterning and generate tissue diversity more reproducibly (Tang‐Schomer *et al*, 2014; Lancaster *et al*, 2016). We anticipate that biochemical and bioengineering advances will rapidly overcome many of the current limitations of cerebral organoids, which are bound to become a standard technique for disease modelling and to provide invaluable insights into molecular and cytoarchitectural orchestration of human brain development.
